# One hundred unanswered questions on the dispersal ecology of fungi

**DOI:** 10.1093/ismejo/wrag018

**Published:** 2026-02-19

**Authors:** Sarah A Cuprewich, Kristin M Barbour, Michelle E Afkhami, Kira M T Lynn, Adriana L Romero-Olivares, Carlos Aguilar-Trigueros, Priscila Chaverri, Cameron P Egan, Veera Norros, Kabir Peay, Robert J Ramos, Ryan Stephens, Lauren Ward, V Bala Chaudhary

**Affiliations:** Environmental Studies, Dartmouth College, Hanover, NH 03755, United States; Ecology, Evolution, Environment and Society Graduate Program, Dartmouth College, Hanover, NH 03755, United States; Department of Ecology and Evolutionary Biology, University of California, Irvine, CA 92697, United States; Department of Biology, University of Miami, Coral Gables, FL 33124, United States; Plant Health Program, Research and Development, Asia Pacific Resources International Holdings Ltd., Pangkalan Kerinci 28300, Riau, Indonesia; Department of Evolution, Ecology and Organismal Biology, University of California, Riverside, CA 92521, United States; Department of Biological and Environmental Science, University of Jyväskylä, Jyväskylä, 40014, Finland; Department of Natural Sciences, Bowie State University, Bowie, MD 20715, United States; Department of Biological Sciences, University of Southern California, Los Angeles, CA 90007, United States; Nature Solutions, Finnish Environment Institute, Helsinki 00790, Finland; Department of Earth System Science, Stanford University, Stanford, CA 94305, United States; Department of Biology, Stanford University, Stanford, CA 94305, United States; Cooperative Institute for Research in Environmental Sciences, University of Colorado, Boulder, CO 80309, United States; Environmental Data Science Innovation & Inclusion Lab, University of Colorado, Boulder, CO 80309, United States; Biological Sciences, East Tennessee State University, Johnson City, TN 37614, United States; Department of Earth System Science, Stanford University, Stanford, CA 94305, United States; Environmental Studies, Dartmouth College, Hanover, NH 03755, United States

**Keywords:** dispersal ecology, dispersal evolution, fungi, spore transport, synthesis, traits

## Abstract

Fungi comprise millions of species that play numerous varied roles in Earth’s natural and managed ecosystems, engaging in a multitude of positive and negative ecological interactions. The dispersal ecology of fungi is central to global biodiversity patterns, maintenance of terrestrial and aquatic ecosystem functions, and tracking human disease and plant pathogen outbreaks. Mycologists have been studying dispersal mechanisms for over a hundred years, but new technology as well as interdisciplinary approaches have reinvigorated research in the field. Here we present 100 research questions in fungal dispersal organized into ten themes: 1) dispersal traits and mechanisms, 2) effects of phenology and lifestyle, 3) spore liberation and transport mechanisms, 4) colonization and establishment, 5) ecosystem-level consequences of dispersal, 6) dispersal in symbiotic and host-associated fungi, 7) dispersal in anthropogenic and changing environments, 8) evolution and tradeoffs in dispersal, 9) role of dispersal in invasion and disease spread, and 10) methodology and techniques. The questions reflect a diversity of new research avenues from fundamental fungal biology to applied ecosystem management and conservation across spatial and temporal scales. They potentially enable integrating fungi and their unique life-history traits and dispersal strategies into existing dispersal frameworks developed around plant and animal systems. We aim to invigorate fungal dispersal research, sparking conversations and providing a focused agenda to widen the tent by illuminating unanswered questions and new research avenues in ecology and evolutionary biology.

## Introduction

Fungi have captured the public’s imagination, playing pivotal roles in our ecosystems, in our croplands, and on our dinner plates [1]. As decomposers, fungi contribute to the flux of nutrients in the environment and carbon storage in soils [2, 3]. Interactions between plants and fungi, ranging from mutualistic to pathogenic, also influence the biogeographic distribution of plant species with cascading effects on biodiversity across trophic levels and ecosystem functioning [4–6]. Further, fungi are linked to human health and agriculture, with fungal pathogens threatening global food security and the spread of fungal diseases expected to increase with ongoing climate change [7–9]. Understanding how fungi move and establish in the environment is essential for predicting disease outbreaks, community assembly, and ecosystem responses to environmental change.

Although researchers have historically focused on how environmental selection influences the spatial distribution of fungal species [10], there are now growing efforts to directly quantify fungal dispersal and identify the mechanisms governing fungal movement [11]. Here we define dispersal as any movement of fungal propagules (e.g. spores, hyphae, cells) from one place to another that has potential consequences for community structure, population dynamics, or evolutionary trajectories (Box 1). However, fungi have unique life forms and life history strategies that make studying fungal dispersal challenging. For instance, fungi can move both actively (hyphal growth, spore ejection, or swimming [12]) and passively (abiotic vectors like air or water or dispersal by animals [13, 14]). Fungi reproduce asexually, sexually, or even through the exchange of nuclei [15, 16]. As research into fungal dispersal has grown, it has highlighted the need for an overarching and collective vision to tackle these fungal-specific challenges and advance the field forward across all stages of dispersal from liberation and transport to deposition and establishment.

Inspired by the flourishing field of movement ecology, the widespread importance of fungi, and the recognition that dispersal of fungi is crucial to predicting ecosystem function in the Anthropocene, we recently assembled the “Fungal Dispersal” Working Group. This working group, which was in the inaugural cohort of workshops at the U.S. National Science Foundation’s (NSF) research center, the Environmental Data Science Innovation & Impact Lab (ESIIL), brings together scientists from 12 academic and government institutions. To ensure diverse and novel perspectives on fungal dispersal, the working group intentionally included scientists from various career stages (e.g. new graduate students to tenured faculty and established government researchers) that are actively pursuing research into the dispersal of fungi in natural [17, 18, 19], [20–25], urban [26, 27], and agricultural [28–30] polar, boreal, temperate, subtropical, tropical, dryland, and mediterranean ecosystems. The group also aimed to include researchers that are tied into the broader ecological and mycological scientific communities (e.g. the Ecological Society of America’s Microbial Ecology section past Chair and a current Student Representative and many active members of the Mycological Society of America). Together, we sought to identify outstanding questions and key research priorities that will help unify and push the field of fungal dispersal ecology forward. These questions are largely not designed to be research questions with reference to specific ecosystems or fungal species; rather, we hope these questions will serve as inspiration for researchers who will tailor them to their system and species of interest. Furthermore, we suggest combining questions to address questions specific to fungal taxonomic groups and/or ecosystems. Here, we present the outcome of this collaboration with the hope that it sparks conversation and motivates meaningful science around fungal dispersal in the next decade.

## Materials and methods

### Participants

The Fungal Dispersal working group, sponsored by ESIIL, convened at the University of Colorado, Boulder for a four day meeting from October 8 to 11, 2024. The working group consisted of 16 participants with expertise in fungal dispersal ecology who were chosen by the lead PI (V.B. Chaudhary) based on three principles: disciplinary diversity, diverse identities, and diverse career stages. Participants work across taxonomic groups and biological scales using different approaches, from mathematical modeling and biophysics to large-scale eDNA sampling surveys, field experiments, and trait based culturing methods in terrestrial, freshwater, and marine ecosystems (albeit the authorship team has less experience with aquatic systems). Together, participants represented a range of career stages including early and late career PhD students, postdoctoral researchers, teaching faculty, assistant, associate, and full professors, and government employees. Together, 12 academic institutions and one government body were represented from three countries (United States of America, Finland, and South Africa). All participants are listed as co-authors.

### Question generation and revision process

Given that identifying research priorities was not the only goal of our working group, we broadly adapted methodology from Sutherland *et al.* [31] to fit within the constraints of our first ESIIL-sponsored group meeting. During the four day meeting, each workshop attendee was asked to generate open questions that, if answered, would considerably impact the field of fungal dispersal ecology. Attendees were asked to create questions that could feasibly be answered by an individual research group or a collaborative program. By the end of the workshop, attendees compiled an initial list of 90 questions.

Workshop participants were then sent an internal survey with a list of the initial 90 questions and asked to rate each question based on the likelihood that answering that research question would move the field of fungal dispersal forward. “Priority” was determined individually by each workshop participant based on discipline, career stage, and personal identity; we did not restrict the proportion of questions that could receive any specific value. Due to a lack of consensus among participants, we could not decisively identify questions that clearly lacked importance and, thus, chose not to remove any questions from the list. A lack of consensus does not indicate that these questions are not important for the field, instead, it highlights the breadth of knowledge yet to be understood and the reality that priorities vary based on region, discipline, and personal identity. Through the survey, workshop participants were also prompted to submit new questions to reflect the disciplinary breadth of fungal dispersal ecology, increasing the collection to 100 questions. Several questions were identified as repetitive during the external review process. Therefore, we took advantage of discussions at our next working group meeting to replace three questions with more unique queries that focus on the importance of interdisciplinarity, promise of technological innovation, and breadth of taxonomic diversity.

**Figure 1:**
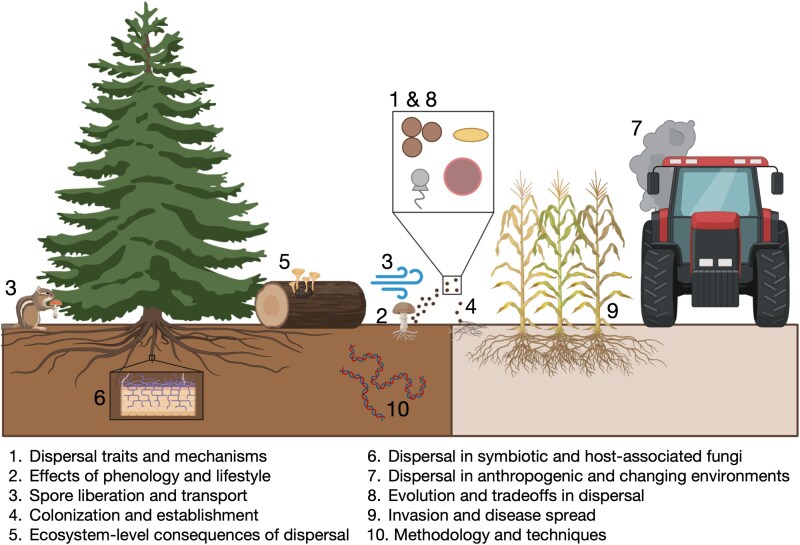
Conceptual diagram displaying each of the ten fungal dispersal themes: 1) dispersal traits and mechanisms, 2) effects of phenology and lifestyle, 3) spore liberation and transport, 4) colonization and establishment, 5) ecosystem-level consequences of dispersal, 6) dispersal in symbiotic and host-associated fungi, 7) dispersal in anthropogenic and changing environments, 8) evolution and tradeoffs in dispersal, 9) invasion and disease spread, and 10) methodology and techniques.

Questions were then screened for duplicates by a subset of attendees, reworded for clarity when needed, and grouped into major themes based on content (Fig. 1). These themes are not meant to reinforce disciplinary divisions or silos, considering many of these questions will require interdisciplinary collaboration, but rather a starting point for further knowledge generation. All authors reviewed and submitted revisions for the final 100 questions prior to manuscript submission.

### Limitations

Although this list of research questions and priorities is not intended to be exhaustive, we acknowledge that the results reflect the interests and expertise of the participants [31]. We would have liked to expand the breadth of our attendees; however, funding restraints limited our capacity to host international researchers without a more substantial travel budget. Some international members were able to participate remotely but differences in time zones and small group discussions restricted their participation compared to in-person attendees. As a result, the majority of workshop participants worked in academic institutions in the United States. Therefore, the questions generated have a geographical bias and may not reflect research priorities of industry or governmental organizations, or those outside of the US. Furthermore, although sorting questions into broad themes is useful to identify research priorities and synergies, they may also place undesired constraints to conceptualizing or approaching open questions.

Although we aim for these 100 questions to be broadly applicable across the kingdom of *Fungi*, the huge diversity of fungal lifestyles and attributes mean that these questions are inherently not exhaustive. However, to help combat any taxonomic biases we suggest basic biological questions comparing dispersal in well characterized groups to groups like early-diverging fungal lineages, which have been broadly under-characterized due to challenges regarding isolation and identification [32]. For example the members of phylum *Neocallimastigomycota* are anaerobic symbionts that live in the digestive tracts of ruminants and other herbivores, an environment that is difficult to replicate in the sterile conditions necessary for isolation and culturing techniques [33]. Aquatic ecosystems are also underrepresented here, which mycologists have long recognized as a bias that is difficult to overcome [34]. Aquatic fungi, including marine fungi, are critically understudied due to challenges in sampling, isolation, and identification yet they are hypothesized to fill various ecological roles from organic matter decomposers to algal parasites [35, 36]. With these biases in mind, we strove to generate questions that are neutral and broadly applicable with respect to specific ecosystems and phylogeny.

## Results

Below, we list all generated questions in ten fungal dispersal themes. The order of questions is not indicative of priority or ease of answering. We introduce each theme via a short introductory paragraph contextualizing the themes within the larger field of fungal dispersal, highlighting current knowledge and key advances of each theme. Lastly, we understand that many of these questions will require interdisciplinary inquiry and thus could have been associated with various themes.

### Dispersal traits and mechanisms

The study of fungal traits is a robust field that seeks to understand how quantifiable characteristics inform key ecological processes like community assembly [37], ecosystem functioning [38], and decomposition [39]. Some recent examples of advances in trait-based fungal ecology include: determining the relationship between spore size and evolutionary trajectory [40], quantifying the effect of symbiotic status on plant resource economics [41], and developing fungal behavior theory within the broader framework of behavioral ecology [42]. Although there have been calls toward functional trait-based studies in fungal ecology [43–45], and large trait databases have been compiled in recent years [46], there is still uncertainty about which traits are most relevant for the study of dispersal due to a lack of correlative and experimental studies directly linking traits with dispersal mechanisms and outcomes [47]. Obtaining a better understanding of fungal traits is necessary because they likely underpin most eco-evolutionary processes, like dispersal, and strengthen our ability to make predictions across highly diverse systems that usually do not share the same fungal taxa. These fungal traits interact with life history strategies to influence dispersal mechanisms, or the modality of movement; outstanding questions about this interaction are highlighted below.

1. What are the primary fungal dispersal mechanisms?
2. How do dispersal strategies vary across ecosystems?
3. When are dispersal traits correlated with habitat or substrate specialization?
4. How do fungi differ in their dispersal rates, distances, modes, and traits?
5. Do spore traits (e.g. size, shape, ornamentation, color) correlate with specific dispersal vectors?
6. Which sporocarp traits (e.g. shape, color, odor, bioluminescence) are adaptive for dispersal?
7. Which sporocarp traits are signals for animal vectors?
8. Which dispersal traits influence fungal species’ invasiveness?
9. How does trait variation change across populations, communities, and ecosystems?
10. How do vertical (up and down within the landscape; y-axis) and horizontal (across the landscape; x-axis) dispersal pathways differ?
11. How do the environmental conditions throughout the process of dispersal influence propagule viability and germination success?
12. Which genes are associated with fungal dispersal traits?
13. What functional genetic variation underpins long-distance dispersal traits in fungi?
14. How does spore or other propagule biochemical composition influence dispersal?
15. How does electric charge and cohesion influence spore or other propagule deposition?

### Effects of phenology and lifestyle

Fungal dispersal is deeply influenced by phenology [48, 49] and the general lifestyle of the fungus, such as whether it is symbiotic or saprotrophic [50] and whether the spores undergo a period of dormancy after release [51]. The timing and environmental triggers of sporocarp and spore production play a key role in determining when and how fungi disperse. The structural adaptations of fungal sporocarps, like spore ornamentation and specialized release mechanisms, correlate with lifestyle and reproductive timing in many groups of fungi [52, 53]. For example, asexual spore production changes across seasons with temperature and relative humidity, which may serve as cues for aerially dispersing fungi to maximize dispersal success [54], whereas symbiotic fungi may exhibit adaptations that facilitate host-to-host transmission [55, 56]. Additionally, knowledge of the sexual cycle may further inform spore longevity and potential for long-distance dispersal [57]. The interplay between phenology, lifestyle, and dispersal strategies is particularly relevant in the context of climate change, as shifting environmental conditions may disrupt established dispersal patterns and affect fungal population dynamics [58, 59]. The following questions investigate how phenological and lifestyle factors shape fungal dispersal across ecosystems.

1. How does the growth stage of a host influence the dispersal of fungal spores or other propagules?
2. What controls the phenology of sporocarp and spore production?
3. How does sporocarp morphology constrain spore morphology and therefore influence dispersal patterns or dispersal efficiency?
4. How might climate change be causing a mismatch between the phenology of sporocarp and spore production?
5. Are fungi optimizing their dispersal by producing sporocarps at specific microsites (e.g. below/within canopy)?
6. How does the geographic distribution of aerial, aquatic, and soil fungi differ?
7. How do dispersal strategies compare among different fungal lifestyles (e.g. biotrophic, pathotrophic, saprotrophic)?
8. How can we study the dispersal of fungi with cryptic sexual cycles?
9. When is it beneficial for fungi to disperse by sexual spores versus asexual spores?
10. What factors determine dormancy duration in fungi, and how do these differences shape dispersal in space and time?

### Spore liberation and transport

The first two stages of the fungal dispersal process are spore (or other propagule) liberation and transport. Liberation refers to the process of becoming physically separated from the fungal body. Then, the spore is transported through biotic or abiotic vectors away from the source across some amount of space. Success in these two stages is critical for establishing a dispersal event. Liberation and transport mechanisms can be active [12] or passive [60], so a variety of methods are usually deployed to quantify these processes in controlled conditions. Evidence for fungal spore liberation strategies can either be observational [13, 61], inferred [26], or experimentally demonstrated [62]. Likewise, spore transport can sometimes be inferred from collecting environmental DNA (eDNA) associated with a potential transport vector [63] or through the release and capture of spore mimics [64]. Parameterizing these first stages of dispersal is crucial for understanding where, when, and how fungal propagules are liberated into and travel around the environment with potential consequences for–for example–plant, animal, and human health.

1. Which factors control the liberation and transport of fungal spores or other propagules (biotic and abiotic)?
2. How do disturbance events influence spore or other propagule liberation?
3. How does human activity impact spore or other propagule liberation mechanisms now and historically?
4. What is the relative importance of primary versus secondary dispersal by animals?
5. Do certain dispersal pathways (e.g. animal-mediated dispersal) result in more directed dispersal than others (e.g. airborne dispersal)?
6. If a fungus can employ multiple dispersal modes, what determines which mode is utilized?
7. When is it generally advantageous for fungi to disperse spores or other propagules near or far from the original source?

### Deposition, establishment, and colonization

Once fungal spores or other propagules are transported to a new location, successful deposition and establishment is the next critical step in the dispersal process [52, 65]. Establishment refers to the initial survival and germination of a spore or other propagule in a new environment, whereas colonization involves subsequent proliferation, resource acquisition, and integration into the microbial community [66]. Although some spores germinate readily under a wide range of conditions, others require specific triggers, such as precise moisture levels, temperature fluctuations, nutrient availability, or even mechanical scarification to initiate growth [66–68]. The establishment of host-associated fungi is often more complex as the state of the host determines if and when a fungus is effectively established (see Theme 6: Dispersal in Symbiotic and Host-Associated Fungi below). The following questions explore the mechanisms that govern fungal establishment and how they influence population persistence, biogeography, and ecosystem dynamics.

1. How do different fungi vary in their ability to establish after dispersal?
2. How do fungal spores or other propagules that fail to germinate contribute to ecosystem dynamics and species interactions?
3. How do biotic and abiotic factors interact to influence spore or other propagule germination and establishment across different fungal taxa?
4. What are the typical establishment rates of fungal spores or other propagules?
5. How does the dispersal of different types of propagules (e.g. spores, hyphae, sclerotia) compare in their contributions to fungal establishment?
6. How does the relative importance of wet versus dry spore deposition vary across environments?
7. What role does plant species composition and canopy structure play in fungal spore or other propagules deposition and establishment?
8. How does spore or other propagule size determine deposition and establishment?
9. How does spore or other propagule density affect establishment success in competitive environments?

### Ecosystem-level consequences of dispersal

Fungal dispersal can have direct and indirect effects on above- and below-ground biodiversity and ecosystem functioning. The various ecological niches fungi occupy translate to supporting a multitude of ecosystem functions, like decomposition [69], nutrient cycling and sequestration [70], and net primary productivity [71]. The ability of fungi to perform these functions depends on their local presence and abundance, often measured at the community scale, which are impacted by dispersal [21]. Fungi also engage in a diversity of ecological interactions, including plants, insects, and other microorganisms. Shifts in fungal composition, diversity, and functioning within an environment can have cascading effects on other species within the community with potential consequences for key terrestrial and aquatic processes [5]. Critically, dispersal limitation can impact fungal diversity in contrasting ways across different spatial scales. For instance, high dispersal limitation can restrict the spread of genetic material, propagules, and/or individuals across a landscape, reducing diversity at the field or landscape scale. In contrast, dispersal limitation can increase regional biodiversity by preventing homogenization of communities. Even though empirical results are contradictory across regional spatial scales, there are theoretical claims that diversity-dispersal relationships are highly nonlinear [72]. Due to the complexity of these relationships, many of the questions below are concerned with investigating properties of communities across scales that may influence ecosystem structure and function. Quantifying the relationship between fungal diversity and ecosystem functioning is in its nascent stages [73]. Thus, developing a deeper understanding of how dispersal contributes to community assembly and functioning will help us disentangle the complexities of fungal diversity-function relationships at the ecosystem scale.

1. How does fungal dispersal contribute to biodiversity and ecosystem assembly?
2. When and where does fungal dispersal most significantly influence ecosystem functioning?
3. When is dispersal a limiting factor for fungal diversity and overall biodiversity within ecosystems?
4. How do fungal dispersal strategies affect competition within ecosystems?
5. What is the relative importance of dispersal versus environmental selection in shaping fungal species ranges?
6. How does fungal dispersal influence the resilience of ecosystems to disturbances?
7. What role does fungal dispersal play in nutrient cycling and soil health?
8. How does fungal dispersal contribute to conservation and restoration efforts of degraded ecosystems?

### Dispersal in symbiotic and host-associated fungi

Symbiotic and other host-associated fungi live in close proximity to their hosts and pose novel challenges in the study of dispersal due to their linked life history trajectories. Phenological mismatches—which occur when once synchronized timing of life events becomes disjointed—have been observed broadly in mutualistic systems [74], studies of movement ecology [75], and predator–prey interactions [76]. Abiotic features of the environment, like timing of snowpack thaw and duration of an annual rainy season, may differentially affect members of a symbiosis with cascading ecosystem effects [25]. However, even though we understand that symbiotic status alters reproductive strategies in fungi, it is unclear how these strategies influence dispersal [40]. Although some symbionts can co-disperse with hosts/partners (e.g. mycorrhizal fungi with plant seeds or lichenized fungi with algae; [77–79]), it remains unclear how common and important codispersal of fungi and their hosts are across the broad biodiversity of the fungal kingdom. These data are essential to parameterize models of host-symbiont dispersal relationships that underpin species distributions. These research questions highlight the importance of quantifying host-associated fungal dispersal for a better understanding of ecosystem functioning.

1. What is the relationship between host and symbiont dispersal?
2. How does seasonal variation in temperature and precipitation affect the dispersal of host-associated fungi?
3. How do free-living and host-associated fungi differ in their dispersal traits?
4. When can the dispersal of symbiotic fungi prime an environment for more successful host dispersal?
5. What are the consequences of spatial or phenological mismatches in the dispersal of symbionts and hosts?
6. How specialized are fungal-animal dispersal interactions?
7. How common is symbiont-host co-dispersal?
8. How do mutualistic fungi adjust their dispersal strategies to match host availability?
9. Under what conditions is optimal dispersal for the symbiont not optimal for the host, leading to conflicting selection pressures on the dispersal of the symbiont-host pair?
10. Are host-associated fungi more or less likely to employ long-distance dispersal mechanisms?
11. Does fungal dispersal cause reproductive changes and/or manipulation of host reproduction and dispersal?

### Dispersal in anthropogenic and changing environments

Climate and other anthropogenic changes to our environment likely affect all stages of the dispersal process. Barring transformative changes to globally dominant economic and political structures, ecosystem organization and function will increasingly be modified by human activities and the ongoing climate crisis. Novel landscapes with increased habitat fragmentation and connectivity enable human-mediated dispersal to occur more often and at a faster rate than previously measured [80]. At the same time, fragmentation of natural habitats can lead to dispersal limitation and population declines [81–83]. Studying dispersal in our changing climate is crucial for predicting future species ranges [84, 85] and management of biodiversity through effective conservation strategies [86]. Additionally, a better understanding of how fungi disperse in the Anthropocene may help curb fungal disease and pathogen outbreaks that impact societies across the globe [87]. Overall, gaining a better understanding of dispersal in anthropogenic and rapidly changing environments is key for maintenance of ecosystem services, improving societal capacity for adaptation and resilience, and building a more sustainable future.

1. How do industrial human activities, like agriculture or deep sea mining, influence fungal community composition and dispersal mechanisms?
2. How does habitat fragmentation affect short and long-distance fungal dispersal?
3. How do soils that are influenced, modified, or created by humans impact fungal dispersal and establishment?
4. How does climate change affect fungal dispersal rates and patterns?
5. How do natural and anthropogenic disturbances differ in their effects on dispersal?
6. When has habitat alteration caused fungi to evolve more effective dispersal traits?
7. How do interspecific differences in dispersal ability explain fungal population trends in human-dominated landscapes?

### Evolution and tradeoffs

Fungi are members of an ancient Kingdom with early diverging lineages that differ substantially from more recently evolved taxa. The evolution of fungal dispersal mechanisms is shaped by a combination of environmental pressures, reproductive strategies, and interactions with hosts or vectors [53, 88, 89]. Fungi have developed diverse dispersal mechanisms, including airborne spores, waterborne propagules, and vector-mediated transmission, to optimize their survival and colonization across a variety of habitats [53, 90]. Occasionally, these mechanisms and associated traits may covary to form a dispersal syndrome with consequences for community structure and ecosystem function [91]. These adaptations come with inherent trade-offs, as the energy invested in spore production, dispersal structures, and survival strategies must be balanced against resource availability and environmental constraints [43, 52, 92]. Furthermore, co-evolution with dispersal vectors, such as animals, can lead to specialized morphological traits, influencing both the efficiency of dispersal and the ability of fungi to adapt to new environments [93]. Understanding these evolutionary dynamics and trade-offs is crucial for elucidating how fungi maintain genetic diversity, respond to ecological pressures, and occupy diverse ecological niches. The questions below explore how dispersal traits evolve and the trade-offs fungi face in optimizing their dispersal strategies.

1. Do early diverging lineages have fundamentally different dispersal mechanisms or traits than later diverging fungal lineages?
2. What drives the evolution of dispersal syndromes?
3. Which fungi exhibit dispersal polymorphisms and why?
4. What is the interspecific variation in spore or other propagule production?
5. Which dispersal traits are adaptive and which are not?
6. What are dispersal-colonization trait tradeoffs?
7. What are the tradeoffs between dispersing via various types of propagules (e.g. spores, sclerotia, hyphae)?
8. What are the tradeoffs between spore size and number within sporocarps (i.e. r- vs k-selected reproductive strategies)?
9. How do fungi balance the energy cost of spore production with survival, colonization success, and dispersal efficiency?
10. What are the tradeoffs between dispersal ability and stress tolerance?
11. How common is coevolution between fungi and their dispersal vectors?
12. What impact does dispersal have on the genetic diversity of fungal populations?
13. How do dispersal rates change across the range of a species?

### Invasion and disease spread

Fungal dispersal is critical to biological invasions and disease dynamics, spreading both beneficial and pathogenic species. A fungus becomes invasive when it expands and establishes beyond its native range, escaping natural ecological constraints [22, 94, 95]. The spread of pathogenic fungi can cause disease outbreaks in agriculture, forestry, and natural ecosystems, often with severe ecological and economic consequences [96]. However, not all fungal invasions are driven by pathogens—many nonpathogenic fungi also invade, often in association with invasive plants, and these co-invasions can have devastating impacts on native biodiversity [97]. Understanding dispersal is essential for predicting and managing fungal invasions and pathogen spread. However, dispersal patterns in managed settings may not fully reflect those in natural ecosystems, where host diversity and environmental variability help maintain ecological balance through co-evolved interactions [95, 98]. Successful invasions depend not only on host susceptibility to pathogens [99] but also on the ability of nonpathogenic fungi to establish in novel environments, outcompete native species, and alter ecological networks [22].

1. How does dispersal ability of a fungus affect its potential role as an invasive species?
2. How do fungi balance rapid dispersal with adaptation to new ecological niches during invasions?
3. How can we leverage fungal dispersal to help control disease spread?
4. When do symbiotic fungi interact with hosts to make invasion more or less successful?
5. How do differences in spore or other propagule dispersal strategies affect the spread of fungal pathogens?
6. What role does dispersal play in the emergence of new fungal diseases?
7. How has human activity changed the geographic distribution and risk of infection for pathogenic fungi?

### Methodology and techniques

Studying fungal dispersal is methodologically challenging due to the microscopic nature of spores and some of the other propagules, complex dispersal pathways, and environmental factors such as wind patterns, humidity, and temperature fluctuations, which influence spore and other propagule transport and deposition [100]. Traditional methods, including field surveys, spore trapping, and culture-based techniques, provide insights into dispersal ecology but often lack resolution [101–103]. Advances in molecular tools, such as real-time polymerase chain reaction (PCR), high-throughput sequencing, and environmental DNA (eDNA) analysis, have improved detection sensitivity and sequencing volume [20, 66, 104, 105]. For example, active aerial eDNA sampling has revealed global patterns of fungal occurrences that shift with space and season [20]. However, biases in sampling, DNA extraction techniques, primer selection, and environmental contamination can affect interpretation [106, 107]. Additionally, DNA-based detection methods do not distinguish between viable and non-viable propagules, which can lead to an overestimation of establishment potential. Integrating viability assays, such as RNA-based detection or propidium monoazide (PMA)-qPCR, can help address this limitation and provide a more accurate understanding of fungal movement [108–110]. Technological fusion from other disciplines (e.g. microfluidic chips for directly observing spore movement) may prove especially promising for overcoming current methodological limitations. However, despite these advancements, challenges remain in standardizing terminology, quantifying the limitations of current approaches, and improving methodological consistency across studies. Addressing these gaps will enhance our ability to track fungal movement and assess its ecological implications.

1. How can we best study fungal dispersal traits in situ?
2. Are novel technologies from movement ecology applicable to fungal dispersal research?
3. How can we coordinate the movement of historically fundamental fungal dispersal research to applied fields?
4. Is our current terminology for fungal dispersal research sufficient to ensure interdisciplinary communication?
5. What frameworks are needed to unify fungal dispersal research across subdisciplines?
6. How can population genetics help us understand fungal dispersal across landscapes?
7. What biases exist in eDNA-based methods (sampling, DNA extraction, primer choice, PCR)?
8. Can we track the dispersal movement of individual fungi from point A to point B using eDNA analysis and is the detection of eDNA sufficient to infer dispersal?
9. How do we measure the role of dispersal in community assembly?
10. What methods are needed to disentangle dispersal limitation from environmental gradients?
11. How do dispersal patterns of viable spores or other propagules differ from eDNA based studies?
12. How can we best facilitate technological transfer and adaptation between disciplines?
13. When is it important to directly measure morphological and biophysical properties of spores versus indirect DNA methods?

## Discussion

Here, we provide a list of 100 unanswered questions aimed to propel the field of fungal dispersal forward so that we can better understand the patterns of fungal diversity, predict ecosystem responses to climate change, and mitigate risks to global human health and food security. Like similar exercises [111–115], this collaborative process allowed us to review the current state of knowledge in this burgeoning discipline and identify key research gaps. The impact of these exercises is also evident. When the findings in Sutherland *et al.* [114] were re-evaluated after 10 years, they found that ample progress was made across research themes but several interdisciplinary priority topics were still underrepresented in the published literature [116]. The diversity of questions and general themes highlight the breadth of research and approaches still needed to understand even the most fundamental aspects of fungal dispersal, and we look forward to revisiting these questions after some time to assess our progress as a field.

Despite the diversity of topics, some common themes were made apparent through this endeavor. For instance, there is a consensus that fungal dispersal research must be integrated across spatial scales, from individuals and populations to ecosystems. Molecular biology may offer tools, like gene editing, to experimentally test hypotheses of which traits are most important for dispersal [117]. Large scale dispersal patterns, which may be identified through spore or eDNA surveying, are underpinned by the morphological, physiological, and behavioral traits of individual species, which are measured through labor-intensive assays in the laboratory. Therefore, answering these questions will require coordinated efforts among disciplines such as fungal biology, evolutionary biology, and community ecology. For example, the unified movement ecology conceptual framework developed by [118] has yet to be applied to microbial systems but may offer key mechanistic insights for fungal dispersal.

Another common thread that emerged is the need to identify trait tradeoffs influencing fungal dispersal. Functional trait tradeoffs, a consequence of finite resources and biological constraints, define niche breadth and life history strategies of species [119]. These tradeoffs also contribute to the maintenance of biodiversity within communities [120, 121]. Assessing how fungal traits vary with dispersal strategies is, thus, necessary to reveal which traits contribute the most to dispersal capability and the context in which specific modes of dispersal are advantageous.

We hope that these questions inspire a unified effort to prioritize and fill in the open knowledge gaps in fungal dispersal. Given the broad ecological, medical, and cultural significance of fungi, it would be beneficial for similar discussions to occur with other diverse perspectives, including the international scientific community, industry researchers (e.g. biotechnology, food and agriculture) policymakers, and the general public. However, spurring action on these research priorities requires investments by funding entities outside their current priorities. For example, assessing the efficacy of current detection methods and development of novel approaches to characterize fungal traits and presence in the field will be essential for continued progress in this research area. With these questions in hand, we aim to spur new frontiers in fungal dispersal ecology and begin tackling growing global challenges in public health, agricultural sustainability, and ecosystem health.

## Data Availability

Data sharing not applicable to this article as no datasets were generated or analyzed during the current study.
